# Biomarkers of sepsis-induced coagulopathy: diagnostic insights and potential therapeutic implications

**DOI:** 10.1186/s13613-025-01434-2

**Published:** 2025-01-17

**Authors:** Anaïs Curtiaud, Toshiaki Iba, Eduardo Angles-Cano, Ferhat Meziani, Julie Helms

**Affiliations:** 1 Faculté de Médecine, Service de Médecine Intensive-Réanimation, Université de Strasbourg (UNISTRA), Hôpitaux universitaires de Strasbourg, Nouvel Hôpital Civil, 1, place de l’Hôpital, Strasbourg, F-67091, cedex France; 2 INSERM (French National Institute of Health and Medical Research), UMR 1260, Regenerative Nanomedicine (RNM), FMTS, Strasbourg, France; 3 Department of Emergency and Disaster Medicine, Juntendo University Graduate School of Medicine, Tokyo, Japan; 4 Innovative Therapies in Haemostasis, Université Paris Cité - INSERM U-1140, Paris, 75006 France

**Keywords:** Disseminated intravascular coagulation, Coagulopathy, Sepsis, Septic shock

## Abstract

Diagnosing coagulopathy in septic patients remains challenging in intensive care. Disseminated intravascular coagulation (DIC) indeed presents with complex pathophysiology, complicating timely diagnosis. Epidemiological data indicate a significant prevalence of DIC in septic patients, with mortality rates up to 60%. Despite advances, current biomarker-based diagnostic tools often fail to provide early and accurate detection. This review evaluates the utility and limitations of traditional and emerging biomarkers for diagnosing sepsis-induced coagulopathy (SIC) and DIC. We also assess the effectiveness of anticoagulant therapy guided by biomarker-based diagnostic criteria.

**Supplementary Information:**

The online version contains supplementary material available at 10.1186/s13613-025-01434-2.

## Introduction

Early diagnosis of coagulopathy in critically ill patients remains a significant challenge in the intensive care setting. Disseminated intravascular coagulation (DIC), depending on its etiology, presents with a complex pathophysiology and heterogeneous phenotypes, complicating timely and accurate diagnosis [1, 2].

Epidemiological data indicate that the frequency of DIC among septic patients varies significantly, with reported rates as high as 40.7% in the KyberSept trial and 22.4% in the PROWESS study [3, 4]. Furthermore, DIC is consistently associated with a high mortality rate, which can reach up to 60% [5, 6], underscoring the need for early diagnosis and intervention. Although our understanding of DIC pathophysiology has improved, current diagnostic tools, primarily based on biomarkers, often fail to provide early and reliable detection. To avoid the misdiagnosis relying on a single biomarker, numerous scoring systems incorporating different hemostasis parameters have been developed.

**Fig. 1:**
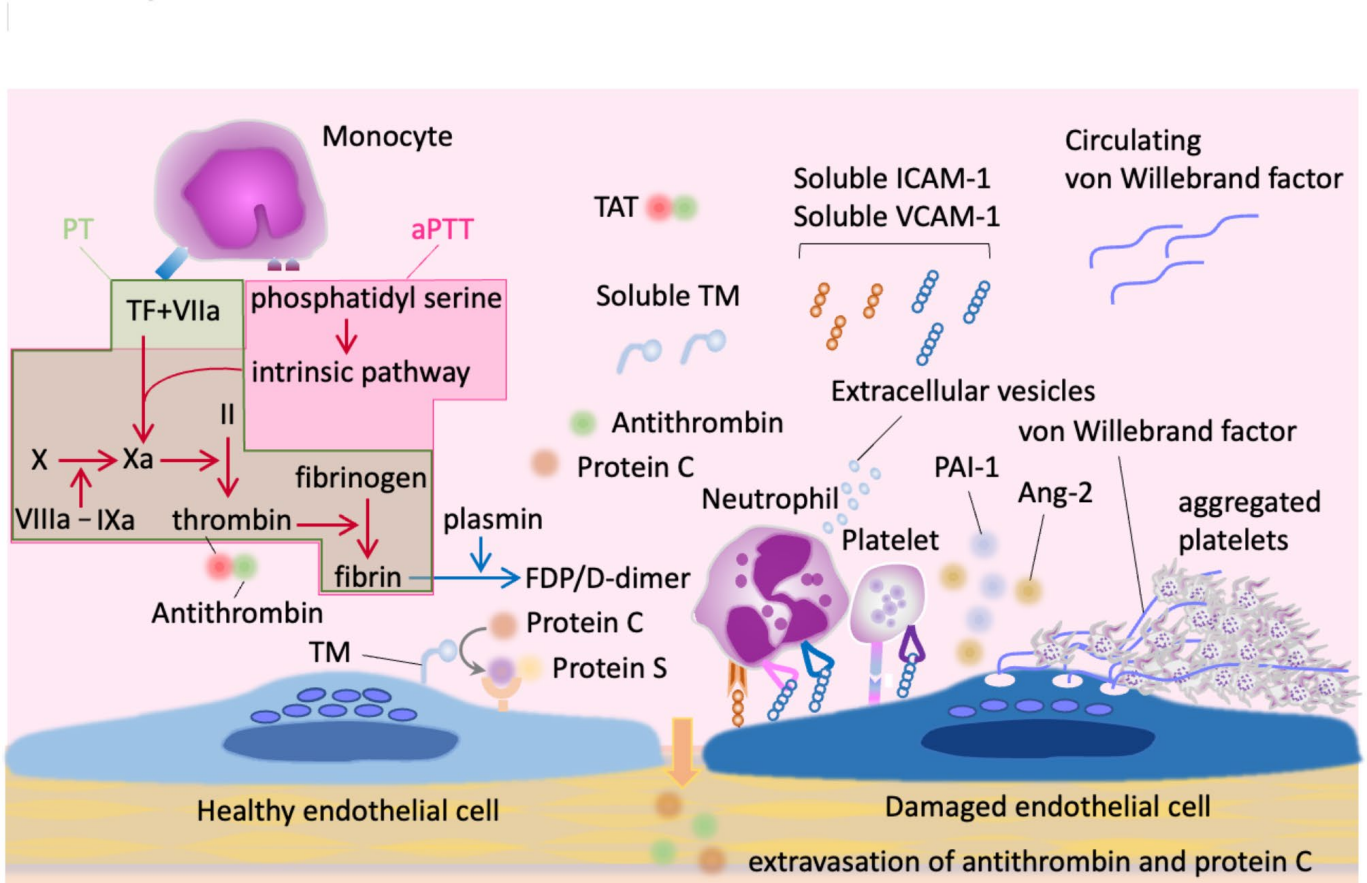
Biomarkers for coagulation disorder in sepsis. Coagulation activation is initiated by tissue factor (TF) and phosphatidylserine expression on monocytes. Other cells, such as platelets, endothelial cells, and granulocytes, also contribute by expressing procoagulant factors. Prothrombin time (PT) and activated partial thromboplastin time (aPTT) tests are commonly used to assess the extrinsic and intrinsic pathways of coagulation. Fibrin, the final coagulation product, is dissolved by plasmin into fibrin/fibrinogen degradation products and D-dimer. Thrombin, a key mediator of intravascular inflammation, is neutralized by antithrombin and converted into an antithrombotic agent by activated protein C. The thrombin generated can be measured as thrombin-antithrombin complex (TAT). Intravascular inflammation promotes interactions between cells, leading to the release of adhesion molecules such as intracellular adhesion molecule-1 (ICAM-1) and vascular cell adhesion molecule-1 (VCAM-1) into the plasma. Additionally, increased levels of plasminogen activator inhibitor-1 (PAI-1), angiopoietin-2 (Ang-2), and von Willebrand factor are known to reflect endothelial damage in sepsis

This review evaluates the utility and limitations of existing biomarkers in diagnosing sepsis-induced coagulopathy (SIC) and overt DIC (Fig. 1). We will first summarize the mechanistic pathways of hemostasis and inflammation involved in DIC. Then, we will highlight biomarkers used for DIC/SIC diagnosis and their diagnostic performances, followed by a discussion on how biomarkers may aid clinicians in treating patients, particularly regarding anticoagulant therapy.

## Mechanistic pathways of hemostasis and inflammation in DIC

In sepsis, DIC arises from a dysregulated immunothrombosis, driven by systemic inflammation and excessive activation of coagulation pathways. The underlying pathophysiological mechanisms encompass a complex interplay between the coagulation cascade, endothelial dysfunction, immune responses and fibrinolytic insufficiency.

### Coagulation activation

The systemic inflammatory response in sepsis leads to the overexpression of tissue factor on activated endothelial cells and monocytes. Tissue factor acts as a potent initiator of the extrinsic coagulation cascade, promoting thrombin generation and resulting in widespread fibrin deposition. Thrombin plays a pivotal role in balancing host defense and pathogenic outcomes during sepsis. Its generation supports pathogen containment through fibrin formation and immune modulation but becomes deleterious when unregulated. Indeed, while localized thrombin activity facilitates pathogen entrapment and immune cell recruitment, systemic thrombin activation contributes to fibrin deposits, microvascular thrombosis, and tissue ischemia [7–9]. Experimental studies highlight that targeted inhibition of thrombin may impair bacterial clearance, underscoring its dual role [10]. Excessive thrombin not only forms fibrin clots but also activates protease-activated receptors on endothelial and immune cells, amplifying inflammation and coagulation activation. This pathological loop underpins the prothrombotic state characteristic of DIC [1, 11] and will be amplified by endothelial dysfunction.

### Endothelial dysfunction

Sepsis induces endothelial cell activation and dysfunction, driven by pro-inflammatory cytokines such as interleukin-6 and tumor necrosis factor-alpha. Endothelial cells lose their anticoagulant properties, including thrombomodulin, and release procoagulant microvesicles (MVs) into the circulation [5, 12]. These MVs enhance thrombin generation and further amplify the coagulation cascade. Increased endothelial permeability due to cytokine-mediated damage leads to vascular leakage, tissue edema, and impaired organ perfusion [13]. While the endothelium plays a central role in amplifying coagulation activation, other vascular cells, including innate immune cells, significantly contribute through the process of immunothrombosis.

### Immunothrombosis dysregulation

Immunothrombosis, initially described as a defense mechanism [14], becomes dysregulated in sepsis. Activated neutrophils release procoagulant MVs, but also neutrophil extracellular traps (NETs), composed of chromatin fibers and granular proteins. While NETs help trap pathogens, they also serve as a scaffold for platelet aggregation and coagulation factor assembly. NETs promote excessive thrombin generation and inhibit fibrinolysis by binding plasminogen, further stabilizing clots [11], which exacerbates the procoagulant environment and contributes to the pathogenesis of DIC.

### Suppressed fibrinolysis

Fibrinolysis, the physiological process responsible for breaking down fibrin clots, is significantly impaired in sepsis-induced DIC. Elevated levels of plasminogen activator inhibitor-1 (PAI-1) released by activated endothelial cells inhibit tissue plasminogen activator (tPA) and urokinase plasminogen activator (uPA), reducing plasmin generation. Furthermore, neutrophil elastase from NETs is responsible for plasminogen degradation, leading to an insufficiency in plasmin generation [15]. As a result, fibrin degradation is hindered, promoting persistent microvascular thrombosis and exacerbating organ dysfunction.

### Microvascular thrombosis and organ failure

The combined effects of excessive coagulation, fibrinolytic suppression, and endothelial dysfunction culminate in widespread microvascular thrombosis. This impairs tissue perfusion, leading to ischemia and driving multi-organ failure, a hallmark of severe DIC [10]. Disseminated microthrombi within critical vascular beds, including the lungs, kidneys, and liver, exacerbate organ dysfunction and increase mortality in septic patients [16].

Understanding these mechanistic pathways underscores the rational for some biomarkers, particularly those associated with initiation of coagulation pathway, the protection of endothelial integrity and the restoration of fibrinolytic balance. Incorporating biomarkers that reflect these processes into clinical practice holds the promise of improving the accuracy of DIC diagnosis and optimizing its management in the context of sepsis.

## Diagnostic biomarkers for DIC and SIC

### Traditional hemostatic markers

Coagulation markers, including traditional markers such as prothrombin time (PT), fibrinogen levels, platelet count, and soluble fibrin monomers, have been fundamental in diagnosing DIC for decades (Supplementary Table 1). These markers are commonly used and are often included in diagnostic scoring systems like those proposed by the International Society on Thrombosis and Haemostasis (ISTH) [17] and the Japanese Association for Acute Medicine (JAAM) [18].

#### Prothrombin time, platelet count, and fibrinogen

PT and platelet count are among the most used markers for assessing coagulation status in critically ill patients. PT evaluates the extrinsic pathway of coagulation and measures the time required for plasma to clot after the addition of thromboplastin, a reagent composed of tissue factor, phospholipids, and calcium. While PT is commonly used to diagnose coagulation disorders and can reflect the consumption of clotting factors when prolonged, its specificity for SIC or DIC is limited, especially in the early stages of these conditions. Studies, such as those by Asakura et al. have shown that while PT is indicative of coagulopathy, it often lacks the specificity needed to differentiate DIC from other conditions, like liver disease or vitamin K deficiency [19].

Similarly, platelet count, which is typically reduced in sepsis-induced DIC due to platelet consumption in the formation of microthrombi and associated to a worse prognosis, also suffers from reduced diagnostic specificity as thrombocytopenia is not exclusive to DIC [20, 21]. In sepsis, other than increased consumption, platelet count can decrease with bone marrow dysfunction. Platelets play a significant role in inflammation and immunity, and their depletion in sepsis often correlates with disease severity and the likelihood of multi-organ failure. Greco et al. have shown that sepsis can lead to a reduction in platelet count as well as impair platelet function, contributing to complications such as multi-organ dysfunction and poor patient outcomes [22]. Additionally, the relationship between platelet count and mean platelet volume (MPV) has been studied as an indicator of bone marrow response in septic patients. A decreasing platelet count and an increasing MPV could be indirect signs of bone marrow activation (?) in the course of sepsis [23]. These findings underscore the importance of monitoring platelet count and function as part of the management and prognosis of sepsis, especially in the context of bone marrow health.

**Fig. 2:**
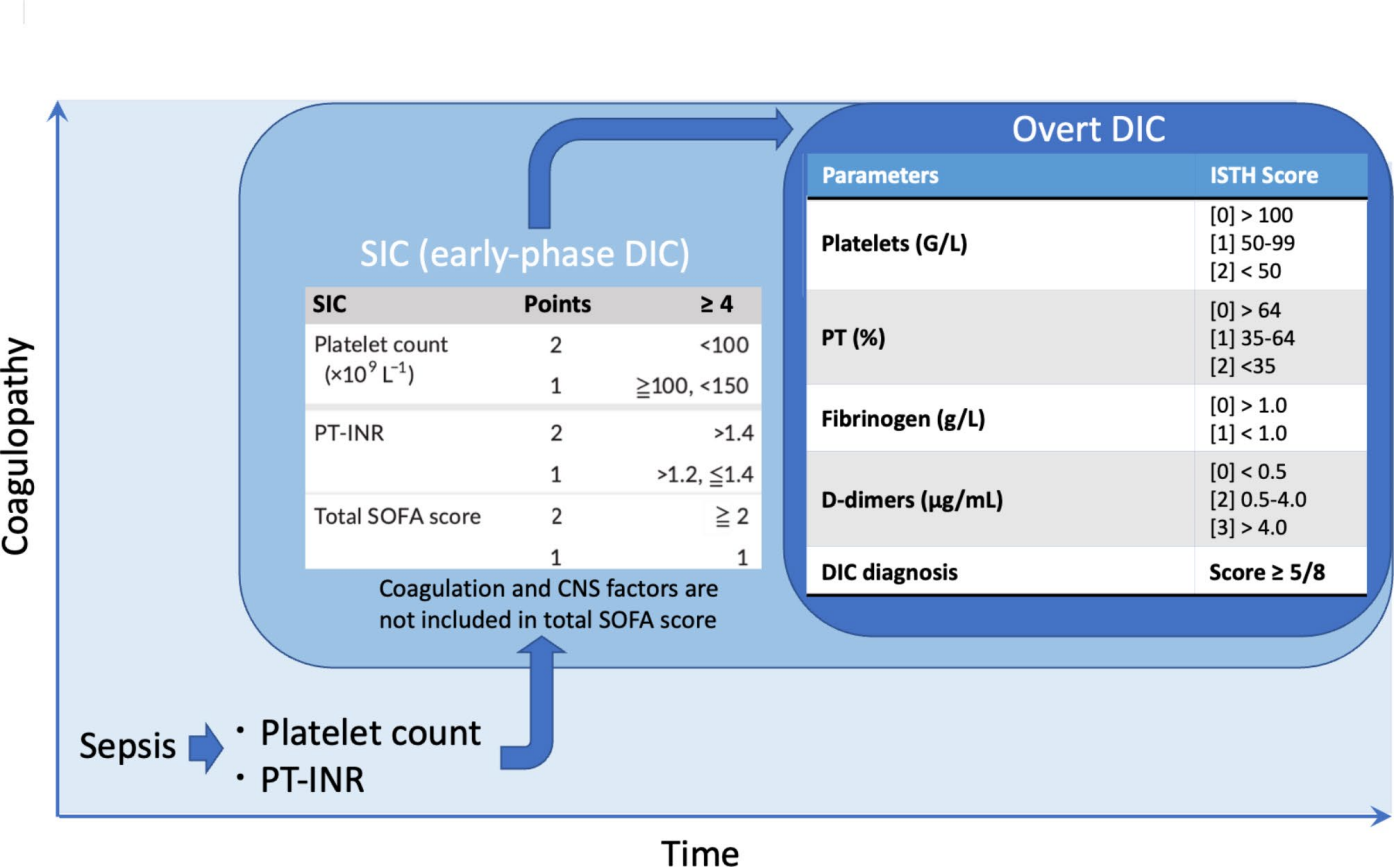
Two step approach to diagnose disseminated intravascular coagulation in sepsis. Coagulation activation is a nearly universal event in sepsis. To screen for coagulation disorders, platelet count and prothrombin time-international normalized ratio (PT-INR) should be measured in all septic patients. If the patient meets the sepsis-induced coagulopathy (SIC) criteria, anticoagulant therapy may be considered. Additionally, fibrinogen and D-dimer levels should be assessed to determine if overt disseminated intravascular coagulation (DIC) is present

Fibrinogen, a key coagulation factor converted to fibrin during clot formation, is generally decreased in DIC due to consumption. However, in sepsis-induced DIC, fibrinogen levels can be paradoxically normal or even elevated [24], complicating the diagnostic process. Individually, each coagulation is limited by issues of sensitivity and specificity which has led to the development of comprehensive scoring systems. The Scientific and Standardization Committee of the ISTH recently introduced the SIC score. This score combines platelet count, PT, and the Sequential Organ Failure Assessment (SOFA) to diagnose DIC at an early stage, before it progresses to overt DIC. The ISTH recommends a two-step diagnostic approach (Fig. 2): first applying the SIC score followed by the overt DIC score if SIC is positive [25]. This approach system represents a significant advance in timely diagnosis without overlooking and may facilitate earlier and more targeted interventions [26].

#### Soluble fibrin and fibrin monomers

Soluble fibrin and fibrin monomers are considered more specific markers of thrombin generation and have been shown to be particularly useful in diagnosing DIC. Wada et al. demonstrated that elevated levels of soluble fibrin are strongly associated with overt DIC, particularly in patients with sepsis, where early diagnosis is critical [27]. However, these markers also suffer from some limitations. Soluble fibrin and fibrin monomers can be elevated in other thrombotic disorders such as myocardial infarction, cancer, and thrombosis. Their measurement is not widely available in all clinical settings and is further hindered by a lack of standardization across laboratories, which significantly limits their practical utility [28, 29].

Emerging evidence suggests that these markers, when used in conjunction with other clinical findings and laboratory tests, can significantly improve the accuracy of DIC diagnosis. For instance, integrating soluble fibrin levels with D-dimer and fibrin/fibrinogen degradation products (FDPs) in a comprehensive scoring system could provide a more nuanced and early diagnosis, particularly in septic patients at high risk for DIC. Elevated levels of soluble fibrin and fibrin monomer indicate ongoing thrombin activity and clot formation, while increases in FDPs and D-dimer also reflect fibrin breakdown. Measuring both offers insight into both the generation and degradation of fibrin, improving diagnostic precision in DIC [30].

### Fibrinolysis markers

Sepsis-induced DIC is characterized by a suppressed fibrinolysis, disrupting the balance between clot formation and its dissolution. This imbalance can lead to excessive clotting, suggesting that fibrinolysis markers might be valuable for diagnosing DIC (Supplementary Table 2).

#### D-dimers and fibrin degradation products

D-dimers, produced from the degradation of fibrin by plasmin, are among the most specific markers of fibrinolysis. Elevated D-dimer levels are a hallmark of DIC and are commonly used in clinical practice to both diagnosing the condition and monitoring its progression. Studies, including those of Watanabe et al. [31] have demonstrated that D-dimer levels are significantly higher in patients with DIC, particularly in those with sepsis-induced coagulopathy.

However, the clinical utility of D-dimers is limited by their lack of specificity. Elevated D-dimer levels can occur in a wide range of conditions, including venous thromboembolism, malignancy, and inflammation, making it challenging to use D-dimers alone as a definitive diagnostic tool for DIC. Furthermore, the suppressed fibrinolysis interferes with the increase of D-dimer, especially in severe cases. Another drawback of D-dimer is the lack of standardization, including significant variations in reference ranges across measurement kits, the absence of a universal threshold, and the use of different monoclonal antibodies, all of which contribute to inconsistent results [27, 32, 33]. It should also be cautioned that D-dimer levels are also affected by age, pregnancy, and other health conditions [34]. As a result, D-dimer levels are typically interpreted alongside other clinical findings and laboratory tests to improve diagnostic accuracy.

FDPs are another marker that reflects fibrinolysis. Like D-dimers, FDPs are elevated in DIC and are often used in combination with other markers in diagnostic scoring systems. FDP levels are generally more sensitive than D-dimer for detecting fibrinolysis and clot degradation, especially in the early stages of DIC. FDP can indicate both fibrin and fibrinogen breakdown, which gives a broader assessment of fibrinolysis compared to D-dimer [35]. Studies have shown that FDPs levels correlate well with disease severity in DIC, with higher levels associated with worse outcomes. However, like D-dimers, FDPs lack specificity and can be elevated in various conditions, highlighting the importance of integrating them into a comprehensive diagnostic approach.

#### Plasmin-α2-antiplasmin complexes

Plasmin-α2-antiplasmin complexes are specific markers of fibrinolytic activity, directly reflecting both the plasmin protein and its active site. When plasmin acts on fibrin, it generates D-dimers, while its interaction with α2-antiplasmin forms an inactive complex. Together, D-dimers and plasmin-α2-antiplasmin offer complementary insights: D-dimers reflect plasmin’s role in fibrin degradation, whereas plasmin-α2-antiplasmin complexes indicate the regulation of plasmin activity, capturing the balance between fibrinolysis and its inhibition. Studies by Wada et al. [36] and others have highlighted the diagnostic value of these complexes in DIC, particularly in patients with hematologic malignancies where fibrinolysis is often upregulated [37]. In DIC, α2-antiplasmin levels are often reduced due to increased consumption, reflecting a compensatory response to ongoing fibrinolysis [19, 38].

While plasmin-α2-antiplasmin complexes are valuable markers of fibrinolytic activity, their levels can be affected by underlying disease and other thrombotic or inflammatory conditions. This variability underscores the importance of interpreting these markers within the broader clinical context.

### Antifibrinolytic markers

Besides α2-antiplasmin, other antifibrinolytic proteins including PAI-1 and thrombin-activatable fibrinolysis inhibitor (TAFI), play critical roles in regulating fibrinolysis and maintaining hemostatic balance. In DIC, particularly sepsis-induced DIC, these proteins are often dysregulated (Supplementary Table 2), contributing to the complex pathophysiology of the condition.

PAI-1 is a potent inhibitor of fibrinolysis, primarily by inhibiting tPA and uPA. Elevated PAI-1 levels are commonly observed in sepsis-induced DIC and are associated with a hypofibrinolytic state, where clot breakdown is impaired. Studies by Asakura et al. [39] and others have shown that high PAI-1 levels correlate with increased mortality in septic patients with DIC, suggesting that PAI-1 could serve as a prognostic marker.

TAFI modulates fibrinolysis by cleaving C-terminal lysine residues on fibrin, thereby inhibiting plasminogen binding and prevents fibrin degradation [40, 41]. The role of TAFI in DIC remains unclear, with studies reporting conflicting results regarding TAFI activity in DIC patients. Some studies suggest that reduced TAFI activity may contribute to hyperfibrinolysis in DIC, while other studies have found no significant differences in TAFI levels between DIC and non-DIC patients [31, 42–44]. These inconsistencies highlight the need for further investigation to determine the role of TAFI in DIC and its potential as a diagnostic or prognostic marker.

Other than PAI-1 and TAFI, circulating DNA released from NETs during sepsis can inhibit fibrinolysis by binding to plasmin and impairing its ability to degrade fibrin. This results in clots that are resistant to breakdown, exacerbating the risk of microvascular thrombosis [45].

### Anticoagulant markers in DIC

The anticoagulant pathways and vascular endothelial function are profoundly affected in DIC, particularly in the context of sepsis. Markers such as antithrombin (AT), protein C, protein S have been extensively studied for their roles in diagnosing and managing DIC (Supplementary Table 3).

#### Antithrombin and protein C systems

AT is a key inhibitor of thrombin and factor Xa, that plays a central role in regulating coagulation. In DIC, AT levels are often reduced due to increased consumption, impaired synthesis, extravasation, and degradation by neutrophil elastase. Several studies, including those by Asakura et al. [19] and Takemitsu et al. [46], have demonstrated that low AT levels are a hallmark of DIC, particularly in sepsis-induced DIC. Low levels of AT in sepsis-associated DIC are highly correlated with increased severity. Studies have shown that AT provides a strong prognostic indicator for the severity of DIC and the likelihood of mortality in patients with sepsis [47]. In addition, combining AT with other markers like thrombin-antithrombin complexes (TAT) improves the diagnostic accuracy for DIC, especially in the early stages, and can help predict the development of overt DIC in septic patients [48]. AT is therefore included in the JAAM-DIC score [49].

The protein C system, which includes protein C and its cofactor protein S, is another essential anticoagulant pathway. Activated protein C (aPC) inactivates factors Va and VIIIa, thereby downregulating thrombin generation [50, 51]. In DIC, the levels of protein C and protein S are often significantly reduced due to consumption and decreased synthesis, contributing to the prothrombotic state observed in these patients [47]. Gando et al. [52] reported that low protein C levels were associated with increased DIC severity and poorer outcomes, highlighting the importance of the protein C pathway in maintaining hemostatic balance.

### Emerging biomarkers

#### Emerging coagulation, inflammation, and endothelial markers

The integration of coagulation markers with biomarkers of inflammation and organ dysfunction is an area of active research (Supplementary Table 4). For example, the combination of PT, soluble fibrin, and fibrin monomers with markers such as C-reactive protein (CRP) or interleukin-6 (IL-6) could potentially enhance the sensitivity and specificity of SIC and DIC diagnosis. Thrombomodulin (TM), and other vascular endothelial markers like microvesicles (MVs) and markers of neutrophil activation like NETs have also been extensively studied for their roles in diagnosing and managing DIC. New biomarkers, such as NETs, MVs and angiopoietin-2 (Ang2), show promise for enhancing the diagnosis of SIC and DIC. However, their widespread clinical adoption faces significant challenges. For some biomarkers, real-time measurement remains constrained by the need for specialized laboratory expertise and advanced technologies, limiting their practicality in time-sensitive clinical settings. Furthermore, their specificity for DIC may be reduced by confounding factors such as systemic inflammation or coexisting conditions, complicating their interpretation.

#### Thrombomodulin and vascular endothelial markers

Thrombomodulin (TM), an endothelial cell membrane protein, plays a crucial role in the activation of protein C. In DIC, endothelial injury causes to the shedding of thrombomodulin from the cell surface, resulting in elevated levels of soluble thrombomodulin (sTM) in the plasma. This increase reflects endothelial dysfunction and disruption in the anticoagulant pathway. Studies by Lin et al. [53] and Zhang et al. [33] have shown that high sTM levels are associated with poor outcomes in DIC, suggesting that sTM could serve as a marker of endothelial damage and a predictor of DIC development in septic patients.

Other vascular endothelial markers, such as MVs and Ang-2, are increasingly recognized for their roles in DIC. MVs, small vesicles released from activated endothelial cells, platelets, and leukocytes, are potent pro-inflammatory and pro-coagulant mediators. Delabranche et al. [5] and Stiel et al. [6] identified elevated levels of endothelial-derived MVs in septic patients with DIC, suggesting their potential as early diagnostic markers. However, the role of MVs in DIC is complex and sometimes contradictory, with some studies reporting decreased MV levels during sepsis [54]. This variability may be due to differences in the cellular origin of MVs or the stage of the disease.

Ang-2, a protein involved in angiogenesis and endothelial activation, is another promising marker. Elevated Ang-2 levels have been associated with endothelial dysfunction and inflammation, making it relevant in the context of DIC. Studies by Statz et al. [55] demonstrated that Ang-2 levels were significantly elevated in DIC patients, suggesting its potential as a marker for monitoring disease progression.

The interactions between platelets, leukocytes, and endothelial cells are vital in determining the severity of sepsis and sepsis-induced DIC [11]. Circulating levels of adhesion molecules are therefore considered to be useful markers for assessing the severity of DIC. Elevated levels of soluble intercellular adhesion molecule-1 (ICAM-1) and soluble vascular cell adhesion molecule-1 (VCAM-1) have been observed in patients with sepsis and are associated with worse clinical outcomes [56]. Although soluble adhesion molecules like ICAM-1 and VCAM-1 are associated with sepsis severity, their sensitivity and specificity for sepsis-induced endothelial damage and organ dysfunction are not high in DIC, and their performance may not be superior to other sepsis biomarkers like procalcitonin or interleukin-6 [57].

#### Neutrophil extracellular traps as a biomarker of DIC

NETs are web-like structures composed of DNA strands and proteins released by neutrophils during a process called NETosis. NETs play a significant role in host defense by trapping and killing pathogens, but in the setting of sepsis-induced DIC, NETs contribute to a procoagulant environment as DNA provides a procoagulant surface for the assembly of coagulation factors (Supplementary Table 5). Delabranche et al. [16] and Stiel et al. [58] have demonstrated that NET formation is increased in septic patients with DIC, promoting thrombin generation and exacerbating coagulopathy. Measuring NETs in a clinical setting, potentially using surrogate markers like neutrophil side scatter fluorescence (NEUT-SFL), could offer a valuable tool for the early diagnosis and management of DIC [59].

## Role of biomarkers in patient management

The diagnostic performance of traditional hemostasis markers alone unfortunately remains limited in the context of sepsis-induced DIC. To address this limitation, several diagnostic scoring systems have been proposed. The ISTH recommends a two-step diagnostic process [25]. The first step involves the SIC score, which combines PT, platelet count, and the SOFA score to identify early coagulopathy. A positive SIC score prompts further evaluation using the ISTH overt DIC scoring system, which integrates platelet count, PT, fibrinogen levels, and fibrin-related markers such as D-dimers or FDPs. A cumulative ISTH score of ≥ 5 confirms overt DIC [25, 26]. This two-step scoring approach aims at facilitating the simple identification of patients with sepsis-induced DIC, allowing for timely intervention [60].

The main limitations of scores are their complexity for bedside application and their inability to facilitate early diagnosis. Also, their utility, and the diagnostic performance of these scores vary. Studies have shown that the ISTH DIC score has good specificity (approximately 91%) but moderate sensitivity (around 50%) for identifying DIC in septic patients [26]. In contrast, the SIC score provides higher sensitivity for early-stage coagulopathy but lower specificity, potentially leading to overdiagnosis in certain contexts like acute on chronic liver failure or treatment by oral anticoagulant. Combining these tools improves diagnostic accuracy by balancing early detection with specificity for overt DIC.

To date, most clinical trials evaluating anticoagulant treatments for sepsis-induced DIC have included patients with sepsis or septic shock regardless of their SIC or DIC status. As a result, based on the prevalence of DIC in septic shock, 60 to 70% of the participants in these trials likely did not meet the criteria for SIC or DIC, potentially diluting the observed treatment effects, as illustrated in the following section. The goal of existing diagnostic scores and novel biomarkers should be to enable the early identification of patients who could benefit from targeted treatment for SIC/DIC.

Heparin is the only anticoagulant widely used for sepsis worldwide. However, the effectiveness of heparin in treating sepsis has not been proven yet and is a subject of ongoing debate. A systematic review and meta-analysis investigating unfractionated or low molecular heparin administered to patients with sepsis, severe sepsis, septic shock, or disseminated intravascular coagulation associated with infection reported that heparin, particularly in low doses, might reduce mortality (Risk Ratio [RR]: 0.88, 95% Confidence Interval [CI]: 0.77-1.00). Meanwhile, heparin was not significantly associated with increased major hemorrhage (RR: 0.79; 95% CI: 0.53–1.17) [61]. While heparin can reduce mortality, it should be cautioned that the effect may be due to lower the risk of thrombosis and not improve coagulation disorder and organ dysfunction. A retrospective analysis - including 3,377 patients with sepsis according to Sepsis 3.0 criteria and a SIC score ≥ 4 - revealed the association between the treatment with unfractionated heparin and decreased intensive care unit mortality in septic patients (Hazard Ratio [HR]: 0.64, 95% CI: 0.49–0.84), and stratification analysis demonstrated the survival advantage was seen only among patients with a SIC score of 4 (HR: 0.56, 95% CI: 0.38–0.81) [62]. Another recent observational study has also reported similar results, and its sub-analysis revealed that significant survival benefits associated with heparin were seen only in the sepsis patients with SIC scores of 3 or 4, and the authors concluded early heparin administration upon admission was associated with lower mortality, especially in moderate SIC without increasing the risk of complications [63]. However, since these results were obtained by the retrospective studies, large clinical trials are needed to confirm the potential benefit of heparin in SIC/DIC and facilitate the development of robust, high-evidence-level recommendations.

While other anticoagulants - such as antithrombin supplementation and recombinant activated protein C - have shown potential benefits in preclinical sepsis / septic shock models, most clinical trials involving patients with sepsis or septic shock, of whom only some had SIC or DIC, failed to demonstrate a survival benefit and were associated with an increased risk of bleeding [64, 65].

Recombinant thrombomodulin (rhTM) has also shown potential for restoring anticoagulant balance and modulating inflammation. SCARLET study is the first international randomized controlled trial comparing rhTM to placebo, including only patients with sepsis-associated coagulopathy [66]. While no significant reduction in 28-day mortality was observed in the overall population, rhTM demonstrated greater effectiveness in subgroups of patients with persistent coagulopathy at the time of the first dose (about 20% had already corrected SIC by the time they received the treatment) and in those not receiving concomitant heparin therapy for thromboprophylaxis. These findings were further supported by a post hoc analysis of the French patient cohort [67]. Despite its promise as a treatment for DIC, larger-scale randomized controlled trials are needed to confirm the efficacy and safety of rhTM in broader septic populations.

In summary, future clinical trials evaluating treatments for SIC/DIC should exclusively enroll patients diagnosed with SIC or DIC, selecting them based on SIC and/or ISTH scoring criteria. Integrating novel biomarkers that reflect underlying pathophysiological processes into clinical practice holds the potential to significantly improve the precision of DIC diagnosis and facilitate the timely initiation of targeted therapies to treat sepsis-induced DIC [1].

## Conclusion

The diagnosis of sepsis-induced DIC remains a significant challenge due to the complex and dynamic nature of the condition. While traditional biomarkers such as PT, platelet count, fibrinogen, and D-dimers, have provided the foundation for DIC diagnosis, their limitations necessitate the development and integration of more specific and sensitive markers. The identification of emerging biomarkers, particularly those reflecting endothelial dysfunction and the anticoagulant pathways, represents a promising avenue for improving diagnostic accuracy and guiding therapeutic interventions, but require further validation. Moreover, real-time measurement of most novel biomarkers remains challenging, as it often requires specialized laboratory expertise and advanced technologies, which currently limit their feasibility in time-sensitive clinical settings.

## Electronic supplementary material

Below is the link to the electronic supplementary material.

Supplementary Material 1

Supplementary Material 2

Supplementary Material 3

Supplementary Material 4

Supplementary Material 5

## Data Availability

NA.
