# Supplementary material for: Biomarkers of sepsis-induced coagulopathy: diagnostic insights and potential therapeutic implications
Source: Ann Intensive Care. 2025 Jan 17;15:12. doi: 10.1186/s13613-025-01434-2 (PMC11739444; doi:10.1186/s13613-025-01434-2)
Supplement: Supplementary file 1 — Supplementary Material 1 [file 13613_2025_1434_MOESM1_ESM.docx]

**Supplementary Table 1. Coagulation markers** **in DIC**

| **Reference** | **Study design** | **Population** | **Markers** | **Results** |
| --- | --- | --- | --- | --- |
| **Meijer et al. 1998**  **(1)** | Case report | 42-year-old woman with a metastatic adenocarcinoma lung cancer | Fibrinogen | Low levels of fibrinogen |
| **Asakura et al. 2001 (2)** | Prospective single center study | 139 patients admitted in ICU for sepsis  68 with DIC  71 without DIC | Platelet count, Fibrinogen, PT | Platelet count and fibrinogen were significantly lower in patient with DIC (p<0.001)  PT: NS |
| **Watanabe et al. 2001 (3)** | Prospective single center study | 114 patients:  36 with DIC*  15 with pre-DIC  63 without DIC  17 healthy volunteers | Fibrinogen | Fibrinogen was significantly lower (p<0.01) in patients with DIC |
| **Wada et al. 2003 (4)** | Prospective single center study | 208 patients with DIC* (ISTH score) | SF | SF was significantly higher in patients with overt-DIC (p<0.01)  ROC analyses: SF was the best marker for DIC diagnosis |
| **Okabayashi et al. 2004 (5)** | Prospective single center study | 1789 patients admitted in the ICU  52 patients with DIC* (ISTH and JMHW criteria) | Platelet count, Fibrinogen, PT | Platelet count and fibrinogen were significantly lower in patients with DIC (p<0.05)  PT and SF were significantly higher (p<0.05) |
| **Wada et al. 2006 (6)** | Prospective single center study | 716 patients  32 with DIC* (ISTH score) | SF | Plasma SF level was significantly higher in patients with DIC  Cut-off value for DIC diagnosis: 8.60 µg/mL |
| **Gando et al. 2007 (7)** | Prospective single center study | 45 patients admitted in ICU for sepsis   11 DIC   34 without DIC | SF | Plasma level of SF was higher in patients with DIC (p<0,01) |
| **Gando et al. 2007 (8)** | Prospective single center study | 48 patients admitted in ICU for sepsis or septic shock  20 patients with DIC  28 without DIC | SF | SF was significantly higher in patients with DIC (p<0.05) |
| **Park et al. 2011 (9)** | Prospective single center study | 139 patients with DIC* (ISTH score)  210 controls | FM | FM was significantly higher in patients with DIC (p<0.0001) and was comparable to DD to diagnose DIC  Cut-off for FM is 7.80 μg/mL |
| **Takemitsu et al. 2011 (10)** | Prospective single center study | 413 patients with diseases associated with DIC*  291 with DIC (JMHW, ISTH, JAAM) | Platelet count, Fibrinogen, FM, PT | Platelet count, FM and fibrinogen were significantly lower in patients with DIC (p<0.01)  The DIC-etiologies were mainly hematologic malignancies  PT was significantly higher in patients with DIC (p<0.01) |
| **Kawasugi**  **et al. 2011 (11)** | Prospective multicentric study | 692 patients, with diseases associated with DIC*  209 patients with DIC (ISTH score) | Platelet count, Fibrinogen, PT, FM | Fibrinogen and platelet count were significantly lower in patients with DIC (p<0.001)  PT and FM were significantly higher in patients with DIC (p<0.001) |
| **Hayakawa et al. 2012 (12)** | Prospective single center study | 50 patients admitted in ICU for sepsis or septic shock  37 patients with DIC  13 without DIC | SF | SF was significantly higher in patients with DIC (p<0.01) |
| **Koyama et al. 2014 (13)** | Prospective single center study | 77 patients admitted in ICU for sepsis   37 DIC (ISTH score)   40 without DIC | Platelet count, Fibrinogen, INR, SF | Platelet count, Fg and SF level were significantly higher in patients with DIC (p=0.036, p=0,041 and p=0.013 respectively)  NS at day 0 between patient with and without DIC for INR, but during first 3 days significant increase (p<0.05) |
| **Masuda et al. 2018 (14)** | Prospective single center study | 107 patients admitted in emergency or ICU for sepsis  42 patients with DIC (JAAM-DIC)  40 patients with pre-DIC  25 without DIC | SF | Plasma SF level was significantly higher in patients with DIC and pre-DIC (p<0.05) |
| **Mei et al. 2018 (15)** | Prospective multicentric study | 444 patients with suspected DIC*  157 overt-DIC (ISTH criteria)  36 pre-DIC  251 without DIC  137 healthy volunteers | Platelet count, Fibrinogen, PT, | Platelet, PT ang fibrinogen were significantly lower in patients with DIC than patients without DIC and between patient with DIC and pre-DIC |
| **Zhang et al. 2021**  **(16)** | Prospective single center study | 172 patients admitted in ICU for sepsis   28 DIC (ISTH score)   144 without DIC | Platelet count, Fibrinogen, PT, | Platelet ang fibrinogen were significantly lower in patients with DIC  PT significantly higher in patients with DIC |

DIC : Disseminated intravascular coagulation ; ICU: intensive care unit ; ISTH : [International Society on Thrombosis and Haemostasis ;](https://www.isth.org/) JAAM : Japanese Association for Acute Medicine ; NS: non statistically significant ; PT: prothrombin time ; SF: soluble fibrin.

*DIC all causes : infection, leukemia, solid cancer, trauma, pregnancy, others

1. Meijer K, Smid WM, Geerards S, van der Meer J. Hyperfibrinogenolysis in disseminated adenocarcinoma. Blood Coagul Fibrinolysis. avr 1998;9(3):279‑83.

2. Asakura H, Ontachi Y, Mizutani T, Kato M, Ito T, Saito M, et al. Depressed plasma activity of plasminogen or á2 plasmin inhibitor is not due to consumption coagulopathy in septic patients with disseminated intravascular coagulation.

3. Watanabe R, Wada H, Watanabe Y, Sakakura M, Nakasaki T, Mori Y, et al. Activity and Antigen Levels of Thrombin-Activatable Fibrinolysis Inhibitor in Plasma of Patients With Disseminated Intravascular Coagulation. Thrombosis Research. oct 2001;104(1):1‑6.

4. Wada H, Sase T, Matsumoto T, Kushiya F, Sakakura M, Mori Y, et al. Increased Soluble Fibrin in Plasma of Patients with Disseminated Intravascular Coagulation. Clin Appl Thromb Hemost. juill 2003;9(3):233‑40.

5. Okabayashi K, Wada H, Ohta S, Shiku H, Nobori T, Maruyama K. Hemostatic markers and the sepsis‐related organ failure assessment score in patients with disseminated intravascular coagulation in an intensive care unit. American J Hematol. juill 2004;76(3):225‑9.

6. Wada H, Kobayashi T, Abe Y, Hatada T, Yamada N, Sudo A, et al. Elevated levels of soluble fibrin or D‐dimer indicate high risk of thrombosis. Journal of Thrombosis and Haemostasis. juin 2006;4(6):1253‑8.

7. Gando S, Hayakawa M, Sawamura A, Hoshino H, Oshiro A, Kubota N, et al. The activation of neutrophil elastase-mediated fibrinolysis is not sufficient to overcome the fibrinolytic shutdown of disseminated intravascular coagulation associated with systemic inflammation. Thrombosis Research. 2007;121(1):67‑73.

8. Gando S, Sawamura A, Hayakawa M, Hoshino H, Kubota N, Nishihira J. High Macrophage Migration Inhibitory Factor Levels in Disseminated Intravascular Coagulation Patients with Systemic Inflammation. Inflammation. 21 juin 2007;30(3‑4):118‑24.

9. Park KJ, Kwon EH, Kim HJ, Kim SH. Evaluation of the Diagnostic Performance of Fibrin Monomer in Disseminated Intravascular Coagulation. Korean J Lab Med. juill 2011;31(3):143‑7.

10. Takemitsu T, Wada H, Hatada T, Ohmori Y, Ishikura K, Takeda T, et al. Prospective evaluation of three different diagnostic criteria for disseminated intravascular coagulation. Thromb Haemost. 2011;105(01):40‑4.

11. Kawasugi K, Wada H, Hatada T, Okamoto K, Uchiyama T, Kushimoto S, et al. Prospective evaluation of hemostatic abnormalities in overt DIC due to various underlying diseases. Thrombosis Research. août 2011;128(2):186‑90.

12. Hayakawa M, Sawamura A, Gando S, Jesmin S, Naito S, Ieko M. A low TAFI activity and insufficient activation of fibrinolysis by both plasmin and neutrophil elastase promote organ dysfunction in disseminated intravascular coagulation associated with sepsis. Thrombosis Research. déc 2012;130(6):906‑13.

13. Koyama K, Madoiwa S, Nunomiya S, Koinuma T, Wada M, Sakata A, et al. Combination of thrombin-antithrombin complex, plasminogen activator inhibitor-1, and protein C activity for early identification of severe coagulopathy in initial phase of sepsis: a prospective observational study. Crit Care. 2014;18(1):R13.

14. Masuda T, Shoko T, Deguchi Y. Clinical Investigation of Coagulation Markers for Early Detection of Sepsis-Induced Disseminated Intravascular Coagulation: A Single-Center, Prospective Observational Study. Clin Appl Thromb Hemost. oct 2018;24(7):1082‑7.

15. Mei H, Jiang Y, Luo L, Huang R, Su L, Hou M, et al. Evaluation the combined diagnostic value of TAT, PIC, tPAIC, and sTM in disseminated intravascular coagulation: A multi-center prospective observational study. Thrombosis Research. janv 2019;173:20‑6.

16. Zhang J, Xue M, Chen Y, Liu C, Kuang Z, Mu S, et al. Identification of soluble thrombomodulin and tissue plasminogen activator-inhibitor complex as biomarkers for prognosis and early evaluation of septic shock and sepsis-induced disseminated intravascular coagulation. Ann Palliat Med. oct 2021;10(10):10170‑84.
