# Supplementary material for: Biomarkers of sepsis-induced coagulopathy: diagnostic insights and potential therapeutic implications
Source: Ann Intensive Care. 2025 Jan 17;15:12. doi: 10.1186/s13613-025-01434-2 (PMC11739444; doi:10.1186/s13613-025-01434-2)
Supplement: Supplementary file 2 — Supplementary Material 2 [file 13613_2025_1434_MOESM2_ESM.docx]

**Supplementary Table 2. Fibrinolysis markers** **in DIC**

| Reference | Study design | Population | Markers | Results |
| --- | --- | --- | --- | --- |
| Plasmin formation | | | | |
| Asakura et al. 1994 (1) | Prospective single center study | 66 patients with DIC* | P-α2APC | P-α2APC was significantly higher in DIC-all causes than healthy volunteers (p<0.01) |
| Wada et al. 2000 (2) | NA | 307 patients with DIC*  121 with non-DIC | P-α2APC | P-α2APC was higher in patients with hematopoietic malignancy than in those without |
| Watanabe et al. 2001 (3) | Prospective single center study | 114 patients  36 with DIC*  15 with pre-DIC  63 without DIC  17 healthy volunteers | P-α2APC | P-α2APC was significantly higher in patients with DIC than in those with non-DIC and healthy control (p<0.01) |
| Okabayashi et al. 2004 (3) | Prospective single center study | 1789 patients admitted in the ICU  52 patients with DIC* | P-α2APC | P-α2APC was significantly higher (p<0.05) in patients with DIC |
| Gando et al. 2009 (4) | Prospective single center study | Patients with and without DIC at an early stage of trauma | P-α2APC | P-α2APC was significantly higher in patients with DIC (p<0.001) |
| Takemitsu et al. 2011 (5) | Prospective single center study | 413 patients with diseases associated with DIC*  291 with DIC | P-α2APC | P-α2APC was significantly (p<0.01), higher in patients with DIC than controls when JAAM criteria was used for DIC diagnosis and NS with ISTH et JMHW scores |
| Kawasugi et al. 2011 (6) | Prospective multicentric study | 692 patients, with diseases associated with DIC*  209 patients with DIC | P-α2APC | P-α2APC was significantly higher in patients with DIC due to HM (and in DIC due to infection disease or solid tumor) than no DIC (p < 0.001) |
| Hayakawa et al. 2012 (7) | Prospective single center study | 50 patients admitted in ICU for sepsis or septic shock  37 patients with DIC  13 without | P-α2APC | P-α2APC was significantly higher in patients with DIC than controls |
| Mei et al. 2018 (8) | Prospective multicentric study | 444 patients with suspected DIC*  157 overt-DIC  36 pre-DIC  251 without DIC  137 healthy volunteers | P-α2APC | P-α2APC was significantly higher in patients with DIC than in those with non-DIC or healthy volunteers (p<0.001) |
| Zhang et al. 2021 (9) | Prospective single center study | 172 patients admitted in ICU for sepsis   28 DIC   144 without DIC | P-α2APC | NS |
| Fibrinolysis markers or plasmin induced secondary fibrinolysis | | | | |
| Watanabe et al. Tr 2001 (3) | Prospective single center study | 114 patients  36 with DIC*  15 with pre-DIC  63 without DIC  17 healthy volunteers | DD | DD were significantly higher in patients with DIC than in those with pre-DIC, non-DIC and healthy volunteers (p<0.01) |
| Wada et al. 2003 (10) | Prospective single center study | 1148 patients  208 patients with overt-DIC*  108 with pre-DIC  770 without DIC | DD, FDP | Plasma level of fibrinolytics markers were significantly higher in patients with overt-DIC (*p < 0.01) |
| Okabayashi et al. 2004 (11) | Prospective single center study | 1789 patients admitted in the ICU  52 patients with DIC* | FDP, DD | FDP and DD were significantly higher (p<0.05) in patients with DIC |
| Wada et al. 2006 (12) | Prospective single center study | 716 patients  32 with DIC* | DD | Plasma DD level was significantly higher in patients with DIC |
| Gando et al. 2007 (13) | Prospective single center study | 45 patients admitted in ICU for sepsis   11 DIC   34 without DIC | DD, FDP | Plasma level of fibrinolytics markers were higher in patients with DIC |
| Park et al. 2011 (14) | Prospective single center study | 139 patients with DIC*  210 controls | DD | DD were significantly higher in patients with DIC (p = 0.0002) |
| Takemitsu et al. 2011 (5) | Prospective single center study | 413 patients with diseases associated with DIC*  291 with DIC | DD, FDP | Plasma level of fibrinolytics markers were higher in patients with DIC (p<0.01) |
| Kawasugi et al. 2011  (6) | Prospective multicentric study | 692 patients, with diseases associated with DIC*  209 patients with DIC | DD, FDP | DD were significantly higher in patients with DIC (p<0.001)  FDP, except in DIC due to solid tumor, was higher but NS |
| Hayakawa et al. 2012 (7) | Prospective single center study | 50 patients admitted in ICU for sepsis or septic shock  37 patients with DIC  13 without | DD  EXDP | DD (p<0.01), EXDP (p<0.05) were significantly higher in patients with DIC than controls |
| Koyama et al. 2014 (15) | Prospective single center study | 77 patients admitted in ICU for sepsis   37 DIC   40 without DIC | FDP, plasminogen | - Plasma FDP level was significantly higher in patients with DIC (p=0.011)  - Plasminogen was significantly lower in patients with DIC (p=0.0001) |
| Asakura et al. 2001 (16) | Prospective single center study | 139 patients admitted in ICU for sepsis  68 with DIC  71 without DIC | FDP, DD, plasminogen | FDP and DD were significantly higher in patients with DIC (p<0.001)  Plasminogen was significantly depressed in patient whit DIC than without (p<0.01) |
| Masuda et al. 2018 (17) | Prospective single center study | 107 patients admitted in emergency or ICU for sepsis  42 patients with DIC  40 patients with pre-DIC  25 without DIC | DD | DD were significantly higher in patients with DIC than pre-DIC and no-DIC group |
| Zhang et al.  2021 (9) | Prospective single center study | 172 patients admitted in ICU for sepsis   28 DIC   144 without DIC | DD | DD were significantly higher in patients with DIC (p<0.001) |
| Antifibrinolytic markers | | | | |
| Asakura et al. 1994 (1) | Prospective single center study | 66 patients with DIC* | PAI-1 | PAI-1 was significantly higher in sepsis DIC than healthy volunteers (p<0.01); no difference between patient with hematologic malignancy or solid tumor DIC and healthy volunteers |
| Meijer et al.  1998 (18) | Case report | 42-year-old woman with a metastatic adenocarcinoma lung cancer | *α*2AP | Low levels of *α*2AP |
| Asakura et al. 2001 (16) | Prospective single center study | 139 patients admitted in ICU for sepsis  68 with DIC  71 without DIC | *α*2AP | *α*2AP was significantly depressed in patient with DIC (p<0.05) |
| Watanabe et al. Tr 2001 (3) | Prospective single center study | 114 patients  36 with DIC*  15 with pre-DIC  63 without DIC  17 healthy volunteers | TAFI activity, TAFI-Ag  PAI-1 | TAFI activity and Ag in plasma were both significantly lower (p<0.01) in patients with DIC than in those with non-DIC and healthy volunteers  PAI-1 was significantly higher in patients with DIC than in those with pre-DIC, non-DIC and healthy control (p<0.01) |
| Chen et al. 2005 (19) | Prospective single center study | 43 patients  20 with overt-DIC*   23 without DIC | TAFI-Ag | NS between two groups |
| Voves et al. 2006 (20) | Prospective single center study | 40 patients admitted in ICU for sepsis   12 with overt-DIC | PAI-1 | PAI-1 was significantly higher in patients with DIC |
| Zeerleder et al. 2006 (21) | Prospective single center study | 40 patients admitted in ICU for septic episode  10 with overt-DIC  30 without DIC | PAI-1, TAFI | PAI-was significantly higher (p=0.008) in patients with DIC  No difference for TAFI between 2 groups |
| Gando et al. 2007 (22) | Prospective single center study | 48 patients admitted in ICU for sepsis or septic shock  20 patients with DIC  28 without DIC | PAI-1 | PAI-1 was significantly higher in patients with DIC (p<0.05) |
| Gando et al. 2007 (13) | Prospective single center study | 45 patients admitted in ICU for sepsis   11 DIC   34 without DIC | PAI-1 | PAI-1 was higher in patients with DIC (p<0.01) |
| Emonts et al. 2008 (23) | Prospective single center study | 112 patients admitted in pediatric ICU for severe meningococcal infection  60 with DIC | TAFI activity | TAFI activity was significantly lower in patients with DIC |
| Gando et al  2009 (4) | Prospective signle center study | Patients with and without DIC at an early stage of trauma | PAI-1 | NS between two groups |
| Hayakawa et al. 2012 (7) | Prospective single center study | 50 patients admitted in ICU for sepsis or septic shock  37 patients with DIC  13 without | PAI-1  TAFI | TAFI was significantly lower in patients with DIC (p<0.05)  PAI-1was significantly higher in patients with DIC |
| Koyama et al. 2014 (15) | Prospective single center study | 77 patients admitted in ICU for sepsis   37 DIC   40 without DIC | PAI-1, *α*2AP | - PAI-1 was higher in patients with DIC (p<0.0001) at day 0  - *α*2AP was lower in patients with DIC (p<0.0001) at day 0  Differences persisted during the first 3 days |
| Hoppensteadt et al. 2015 (24) | Multicenter randomized trial  ART-123 (25) | 750 patients randomized with sepsis  617 plasma samples  98 overt-DIC  30 healthy volunteers | PAI-1 | PAI-1 was higher in patient with overt-DIC than healthy volunteers |
| Masuda et al. 2018 (17) | Prospective single center study | 107 patients admitted in emergency or ICU for sepsis  42 patients with DIC  40 patients with pre-DIC  25 without DIC | PAI-1 | PAI-1 was significantly higher in patients with DIC and pre-DIC than patients without DIC |
| Mei et al. 2018 (8) | Prospective multi-center study | 444 patients with suspected DIC*  157 overt-DIC  36 pre-DIC  251 without DIC  137 healthy volunteers | PAI-1 | PAI-1was significantly higher in patients with DIC than non-DIC and controls (p<0.001) |
| Zhang et al.  2021 (9) | Prospective single center study | 172 patients admitted in ICU for sepsis   28 DIC  144 without DIC | PAI-1 | PAI-1was significantly higher in patients with DIC than controls (p<0.001) |

Ag: antigen; *α*2AP: *α*2-antiplasmin; DD: D-dimer; DIC: disseminated intravascular coagulation; EXDP: elastase-mediated fibrin degradation products; FDPs: fibrinogen degradation products; FM: fibrin monomer; FRMs: fibrin-related markers; HM: hematopoietic malignancy; ISTH : [International Society on Thrombosis and Haemostasis ;](https://www.isth.org/) NA : not applicable ; NS: non statistically significant ; PAI-1: plasminogen activator inhibitor-1; P-α2APC: inactive plasmin-α2AP complex; SF: soluble fibrin.

*DIC all causes: infection, leukemia, solid cancer, trauma, pregnancy, others

1. Study of the balance between coagulation and fibrinolysis in disseminated intravascular coagulation using molecular markers. Asakura et al Blood 1994.

2. Wada H, Gabazza E, Nakasaki T, Shimura M, Hiyoyama K, Deguchi K, et al. Diagnosis of Disseminated Intravascular Coagulation by Hemostatic Molecular Markers. Seminars in Thrombosis and Hemostasis. 31 déc 2000;26:17‑22.

3. Watanabe R, Wada H, Watanabe Y, Sakakura M, Nakasaki T, Mori Y, et al. Activity and Antigen Levels of Thrombin-Activatable Fibrinolysis Inhibitor in Plasma of Patients With Disseminated Intravascular Coagulation. Thrombosis Research. oct 2001;104(1):1‑6.

4. Gando S. Acute Coagulopathy of Trauma Shock and Coagulopathy of Trauma: A Rebuttal. You Are Now Going Down the Wrong Path. Journal of Trauma: Injury, Infection & Critical Care. août 2009;67(2):381‑3.

5. Takemitsu T, Wada H, Hatada T, Ohmori Y, Ishikura K, Takeda T, et al. Prospective evaluation of three different diagnostic criteria for disseminated intravascular coagulation. Thromb Haemost. 2011;105(01):40‑4.

6. Kawasugi K, Wada H, Hatada T, Okamoto K, Uchiyama T, Kushimoto S, et al. Prospective evaluation of hemostatic abnormalities in overt DIC due to various underlying diseases. Thrombosis Research. août 2011;128(2):186‑90.

7. Hayakawa M, Sawamura A, Gando S, Jesmin S, Naito S, Ieko M. A low TAFI activity and insufficient activation of fibrinolysis by both plasmin and neutrophil elastase promote organ dysfunction in disseminated intravascular coagulation associated with sepsis. Thrombosis Research. déc 2012;130(6):906‑13.

8. Mei H, Jiang Y, Luo L, Huang R, Su L, Hou M, et al. Evaluation the combined diagnostic value of TAT, PIC, tPAIC, and sTM in disseminated intravascular coagulation: A multi-center prospective observational study. Thrombosis Research. janv 2019;173:20‑6.

9. Zhang J, Xue M, Chen Y, Liu C, Kuang Z, Mu S, et al. Identification of soluble thrombomodulin and tissue plasminogen activator-inhibitor complex as biomarkers for prognosis and early evaluation of septic shock and sepsis-induced disseminated intravascular coagulation. Ann Palliat Med. oct 2021;10(10):10170‑84.

10. Wada H, Sase T, Matsumoto T, Kushiya F, Sakakura M, Mori Y, et al. Increased Soluble Fibrin in Plasma of Patients with Disseminated Intravascular Coagulation. Clin Appl Thromb Hemost. juill 2003;9(3):233‑40.

11. Okabayashi K, Wada H, Ohta S, Shiku H, Nobori T, Maruyama K. Hemostatic markers and the sepsis‐related organ failure assessment score in patients with disseminated intravascular coagulation in an intensive care unit. American J Hematol. juill 2004;76(3):225‑9.

12. Wada H, Kobayashi T, Abe Y, Hatada T, Yamada N, Sudo A, et al. Elevated levels of soluble fibrin or D‐dimer indicate high risk of thrombosis. Journal of Thrombosis and Haemostasis. juin 2006;4(6):1253‑8.

13. Gando S, Hayakawa M, Sawamura A, Hoshino H, Oshiro A, Kubota N, et al. The activation of neutrophil elastase-mediated fibrinolysis is not sufficient to overcome the fibrinolytic shutdown of disseminated intravascular coagulation associated with systemic inflammation. Thrombosis Research. 2007;121(1):67‑73.

14. Park KJ, Kwon EH, Kim HJ, Kim SH. Evaluation of the Diagnostic Performance of Fibrin Monomer in Disseminated Intravascular Coagulation. Korean J Lab Med. juill 2011;31(3):143‑7.

15. Koyama K, Madoiwa S, Nunomiya S, Koinuma T, Wada M, Sakata A, et al. Combination of thrombin-antithrombin complex, plasminogen activator inhibitor-1, and protein C activity for early identification of severe coagulopathy in initial phase of sepsis: a prospective observational study. Crit Care. 2014;18(1):R13.

16. Asakura H, Ontachi Y, Mizutani T, Kato M, Ito T, Saito M, et al. Depressed plasma activity of plasminogen or á2 plasmin inhibitor is not due to consumption coagulopathy in septic patients with disseminated intravascular coagulation.

17. Masuda T, Shoko T, Deguchi Y. Clinical Investigation of Coagulation Markers for Early Detection of Sepsis-Induced Disseminated Intravascular Coagulation: A Single-Center, Prospective Observational Study. Clin Appl Thromb Hemost. oct 2018;24(7):1082‑7.

18. Meijer K, Smid WM, Geerards S, van der Meer J. Hyperfibrinogenolysis in disseminated adenocarcinoma. Blood Coagul Fibrinolysis. avr 1998;9(3):279‑83.

19. Chen CC, Lee KD, Gau JP, Yu YB, You JY, Lee SC, et al. Plasma antigen levels of thrombin-activatable fibrinolysis inhibitor did not differ in patients with or without disseminated intravascular coagulation. Ann Hematol. oct 2005;84(10):675‑80.

20. Voves C, Wuillemin WA, Zeerleder S. International Society on Thrombosis and Haemostasis score for overt disseminated intravascular coagulation predicts organ dysfunction and fatality in sepsis patients. Blood Coagulation & Fibrinolysis. sept 2006;17(6):445‑51.

21. Zeerleder S, Schroeder V, Hack CE, Kohler HP, Wuillemin WA. TAFI and PAI-1 levels in human sepsis. Thrombosis Research. janv 2006;118(2):205‑12.

22. Gando S, Sawamura A, Hayakawa M, Hoshino H, Kubota N, Nishihira J. High Macrophage Migration Inhibitory Factor Levels in Disseminated Intravascular Coagulation Patients with Systemic Inflammation. Inflammation. 21 juin 2007;30(3‑4):118‑24.

23. Emonts M, De Bruijne ELE, Guimarães AHC, Declerck PJ, Leebeek FWG, De Maat MPM, et al. Thrombin activatable fibrinolysis inhibitor is associated with severity and outcome of severe meningococcal infection in children. Journal of Thrombosis and Haemostasis. févr 2008;6(2):268‑76.

24. Hoppensteadt D, Tsuruta K, Hirman J, Kaul I, Osawa Y, Fareed J. Dysregulation of Inflammatory and Hemostatic Markers in Sepsis and Suspected Disseminated Intravascular Coagulation. Clin Appl Thromb Hemost. mars 2015;21(2):120‑7.

25. Vincent JL, Ramesh MK, Ernest D, LaRosa SP, Pachl J, Aikawa N, et al. A Randomized, Double-Blind, Placebo-Controlled, Phase 2b Study to Evaluate the Safety and Efficacy of Recombinant Human Soluble Thrombomodulin, ART-123, in Patients With Sepsis and Suspected Disseminated Intravascular Coagulation*: Critical Care Medicine. sept 2013;41(9):2069‑79.
