# Supplementary material for: Biomarkers of sepsis-induced coagulopathy: diagnostic insights and potential therapeutic implications
Source: Ann Intensive Care. 2025 Jan 17;15:12. doi: 10.1186/s13613-025-01434-2 (PMC11739444; doi:10.1186/s13613-025-01434-2)
Supplement: Supplementary file 3 — Supplementary Material 3 [file 13613_2025_1434_MOESM3_ESM.docx]

**Table 3. Anticoagulant markers in DIC**

| Reference | Study design | Population | Markers | Results |
| --- | --- | --- | --- | --- |
| Meijer et al.  1998 (9) | Case report | 42-year-old woman with a metastatic adenocarcinoma lung cancer | AT, pC, pS | Normal levels of AT, pC and pS |
| Asakura et al. 2001 (10) | Prospective single center study | 139 patients admitted in ICU for sepsis  68 with DIC  71 without DIC | AT | AT was significantly depressed in patient whit DIC than without (p<0.001) |
| Okabayashi et al 2004 (11) | Prospective single center study | 1789 patients admitted in the ICU  52 patients with DIC | AT | TM was NS between DIC and no-DIC patients  AT was significantly lower in patients with DIC (p<0.05) |
| Gando et al. 2007(2) | Prospective single center study | 45 patients admitted in ICU for sepsis   11 DIC   34 without DIC | AT, pC | AT and pC were lower (p<0.05) in patient with DIC than without |
| Gando et al. 2007 (12) | Prospective single center study | 48 patients admitted in ICU for sepsis or septic shock  20 patients with DIC  28 without DIC | pC | pC was lower (p<0.05) in patient with DIC than without |
| Takemitsu et al. 2011 (4) | Prospective single center study | 413 patients with diseases associated with DIC*  291 with DIC | AT | AT was lower (p<0.01) in patient with DIC |
| Kawasugi et al. (5) | Prospective multicentric study | 692 patients, with diseases associated with DIC*  209 patients with DIC diagnose by ISTH criteria | AT | AT was lower in patient with sepsis-induced DIC (p<0.01) but NS in DIC due to solid tumor or hematologic malignancy |
| Koyama et al. 2014 (6) | Prospective single center study | 77 patients admitted in ICU for sepsis   37 DIC   40 without DIC | AT, pC | AT and pC were lower (p<0.0001) in patient with DIC at day 0 |
| Choi et al.  2014 (7) | NA | 126 patients  55 overt-DIC* | AT, pC, pS | pC (p<0.01), AT and pS were lower (p < 0.05) in patient with DIC |
| Hoppensteadt et al. 2015 (13) | Multicenter randomized trial  ART-123 | 750 patients with sepsis  617 plasma samples  98 overt-DIC  30 HV | pC | pC was lower in patient with overt-DIC and non-overt DIC than HV |

AT: antithrombin; DIC: disseminated intravascular coagulation; NA: not applicable; NS: non statistically significant; pC: protein C; pS: protein S

*DIC all causes: infection, leukemia, solid cancer, trauma, pregnancy, others

1. Watanabe R, Wada H, Watanabe Y, Sakakura M, Nakasaki T, Mori Y, et al. Activity and Antigen Levels of Thrombin-Activatable Fibrinolysis Inhibitor in Plasma of Patients With Disseminated Intravascular Coagulation. Thrombosis Research. oct 2001;104(1):1‑6.

2. Gando S, Hayakawa M, Sawamura A, Hoshino H, Oshiro A, Kubota N, et al. The activation of neutrophil elastase-mediated fibrinolysis is not sufficient to overcome the fibrinolytic shutdown of disseminated intravascular coagulation associated with systemic inflammation. Thrombosis Research. 2007;121(1):67‑73.

3. Lin SM, Wang YM, Lin HC, Lee KY, Huang CD, Liu CY, et al. Serum thrombomodulin level relates to the clinical course of disseminated intravascular coagulation, multiorgan dysfunction syndrome, and mortality in patients with sepsis*: Critical Care Medicine. mars 2008;36(3):683‑9.

4. Takemitsu T, Wada H, Hatada T, Ohmori Y, Ishikura K, Takeda T, et al. Prospective evaluation of three different diagnostic criteria for disseminated intravascular coagulation. Thromb Haemost. 2011;105(01):40‑4.

5. Kawasugi K, Wada H, Hatada T, Okamoto K, Uchiyama T, Kushimoto S, et al. Prospective evaluation of hemostatic abnormalities in overt DIC due to various underlying diseases. Thrombosis Research. août 2011;128(2):186‑90.

6. Koyama K, Madoiwa S, Nunomiya S, Koinuma T, Wada M, Sakata A, et al. Combination of thrombin-antithrombin complex, plasminogen activator inhibitor-1, and protein C activity for early identification of severe coagulopathy in initial phase of sepsis: a prospective observational study. Crit Care. 2014;18(1):R13.

7. Choi Q, Hong KH, Kim JE, Kim HK. Changes in Plasma Levels of Natural Anticoagulants in Disseminated Intravascular Coagulation: High Prognostic Value of Antithrombin and Protein C in Patients with Underlying Sepsis or Severe Infection. Ann Lab Med. 1 mars 2014;34(2):85‑91.

8. Zhang J, Xue M, Chen Y, Liu C, Kuang Z, Mu S, et al. Identification of soluble thrombomodulin and tissue plasminogen activator-inhibitor complex as biomarkers for prognosis and early evaluation of septic shock and sepsis-induced disseminated intravascular coagulation. Ann Palliat Med. oct 2021;10(10):10170‑84.

9. Meijer K, Smid WM, Geerards S, van der Meer J. Hyperfibrinogenolysis in disseminated adenocarcinoma. Blood Coagul Fibrinolysis. avr 1998;9(3):279‑83.

10. Asakura H, Ontachi Y, Mizutani T, Kato M, Ito T, Saito M, et al. Depressed plasma activity of plasminogen or á2 plasmin inhibitor is not due to consumption coagulopathy in septic patients with disseminated intravascular coagulation.

11. Okabayashi K, Wada H, Ohta S, Shiku H, Nobori T, Maruyama K. Hemostatic markers and the sepsis‐related organ failure assessment score in patients with disseminated intravascular coagulation in an intensive care unit. American J Hematol. juill 2004;76(3):225‑9.

12. Gando S, Sawamura A, Hayakawa M, Hoshino H, Kubota N, Nishihira J. High Macrophage Migration Inhibitory Factor Levels in Disseminated Intravascular Coagulation Patients with Systemic Inflammation. Inflammation. 21 juin 2007;30(3‑4):118‑24.

13. Hoppensteadt D, Tsuruta K, Hirman J, Kaul I, Osawa Y, Fareed J. Dysregulation of Inflammatory and Hemostatic Markers in Sepsis and Suspected Disseminated Intravascular Coagulation. Clin Appl Thromb Hemost. mars 2015;21(2):120‑7.
