# Supplementary material for: Biomarkers of sepsis-induced coagulopathy: diagnostic insights and potential therapeutic implications
Source: Ann Intensive Care. 2025 Jan 17;15:12. doi: 10.1186/s13613-025-01434-2 (PMC11739444; doi:10.1186/s13613-025-01434-2)
Supplement: Supplementary file 4 — Supplementary Material 4 [file 13613_2025_1434_MOESM4_ESM.docx]

**Supplementary Table 4. Emerging coagulation and vascular cell markers**

| Reference | Study design | Population | Markers type | Results |
| --- | --- | --- | --- | --- |
| Watanabe et al. 2001(1) | Prospective single center study | 114 patients:  36 with DIC*  15 with pre-DIC  63 without DIC  17 HV | TM | TM was significantly higher in patients with DIC than in those with non-DIC and healthy control (p<0.01) |
| Okabayashi et al 2004 (2) | Prospective single center study | 1789 patients admitted in the ICU  52 patients with DIC* | TM | TM was NS between DIC and no-DIC patients |
| Lin et al. 2008 (3) | Prospective single center study | 100 patients admitted in ICU for sepsis  30 patients with DIC | sTM | sTM was significantly higher in patients with DIC (p<0.05) |
| Takemitsu et al. 2011 (4) | Prospective single center study | 413 patients with diseases associated with DIC*  291 with DIC | TM | TM was significantly higher in patients with DIC (p<0.001) |
| Kawasugi et al. 2011 (5) | Prospective multicentric study | 692 patients, with diseases associated with DIC*  209 patients with DIC | TM | TM was significantly lower in patient with sepsis-induced DIC than without (p<0.01) but NS in DIC due to solid tumor or hematologic malignancy |
| Delabranche et al. 2013 (6) | Prospective multicenter study | 100 patients with septic shock  40 DIC  60 without DIC | MVs  sE-selectin | Endothelium-derived CD105-MVs and CD31-MVs were associated with early DIC (p<0.05)  sE-selectin was higher in DIC patients (p<0.01) |
| Delabranche et al. 2016 (7) | Prospective multicenter study (validation cohort) | 265 patients with septic shock  93 DIC  172 without DIC | MVs | Endothelium-derived CD105-MVs were associated with early DIC (p<0.05) |
| Stiel et al. 2016 (8) | Prospective single center study | 100 patients with septic shock  35 patients with DIC | MVs | Significant elevation of CD66b-MVs in DIC patients |
| Mei et al. 2018 (9) | Prospective multi-center study | 444 patients with suspected DIC*  157 overt-DIC  36 pre-DIC  251 without DIC  137 HV | sTM | sTM was significantly higher in patients with DIC (p<0.001) |
| Statz et al.  2018 (10) | Prospective single center study | 101 patients admitted in ICU for sepsis   24 DIC | Ang-2 | Ang-2 was significantly higher in patient with DIC at day 0, 4 and 8 |
| Walborn et al. 2019 (11) | Prospective single center study | 103 patients admitted in ICU for sepsis   24 DIC | Ang-2 | Ang-2 was significantly higher in patient with DIC (p<0.05) |
| Higgins et al. 2018 (12) | Prospective cohort study | Sample of patients admitted in ICU for sepsis | Ang-2 | Ang-2 was significantly higher in patient with sepsis-DIC (p<0.005)  Cut-off value: 10 ng/ml |
| Boscolo et al. 2019 (13) | Case-control study | 80 patients  40 HV  40 patients with septic shock  12 DIC | CD61-MVs (platelet-derived MP) | Ratio PMP/Plts significantly higher in DIC group vs. non-DIC (p<0.025) |
| Zhang et al.  2021(14) | Prospective single center study | 172 patients admitted in ICU for sepsis   28 DIC   144 without DIC | sTM | sTM was significantly higher in patients with DIC than controls (p<0.001) |
| Wegrzyn et al. 2021 (15) | Prospective single center study | 103 patients admitted in ICU for sepsis | MVs-TF | Significantly higher in sepsis with DIC group vs HV |

Ang-2: angiopoietin 2 ; DIC: disseminated intravascular coagulation; HV: healthy volunteers ; MVs: microvesicles; NS: non statistically significant; plts: platelets; sTM: thrombomodulin; TM: thrombomodulin; TF: tissue factor.

*DIC all causes: infection, leukemia, solid cancer, trauma, pregnancy, others.

1. Watanabe R, Wada H, Watanabe Y, Sakakura M, Nakasaki T, Mori Y, et al. Activity and Antigen Levels of Thrombin-Activatable Fibrinolysis Inhibitor in Plasma of Patients With Disseminated Intravascular Coagulation. Thrombosis Research. oct 2001;104(1):1‑6.

2. Okabayashi K, Wada H, Ohta S, Shiku H, Nobori T, Maruyama K. Hemostatic markers and the sepsis‐related organ failure assessment score in patients with disseminated intravascular coagulation in an intensive care unit. American J Hematol. juill 2004;76(3):225‑9.

3. Lin SM, Wang YM, Lin HC, Lee KY, Huang CD, Liu CY, et al. Serum thrombomodulin level relates to the clinical course of disseminated intravascular coagulation, multiorgan dysfunction syndrome, and mortality in patients with sepsis*: Critical Care Medicine. mars 2008;36(3):683‑9.

4. Takemitsu T, Wada H, Hatada T, Ohmori Y, Ishikura K, Takeda T, et al. Prospective evaluation of three different diagnostic criteria for disseminated intravascular coagulation. Thromb Haemost. 2011;105(01):40‑4.

5. Kawasugi K, Wada H, Hatada T, Okamoto K, Uchiyama T, Kushimoto S, et al. Prospective evaluation of hemostatic abnormalities in overt DIC due to various underlying diseases. Thrombosis Research. août 2011;128(2):186‑90.

6. Delabranche X, Boisramé-Helms J, Asfar P, Berger A, Mootien Y, Lavigne T, et al. Microparticles are new biomarkers of septic shock-induced disseminated intravascular coagulopathy. Intensive Care Med. oct 2013;39(10):1695‑703.

7. Delabranche X, Quenot JP, Lavigne T, Mercier E, François B, Severac F, et al. Early Detection of Disseminated Intravascular Coagulation During Septic Shock: A Multicenter Prospective Study. Critical Care Medicine. oct 2016;44(10):e930‑9.

8. Stiel L, Delabranche X, Galoisy AC, Severac F, Toti F, Mauvieux L, et al. Neutrophil Fluorescence: A New Indicator of Cell Activation During Septic Shock–Induced Disseminated Intravascular Coagulation. Critical Care Medicine. nov 2016;44(11):e1132‑6.

9. Mei H, Jiang Y, Luo L, Huang R, Su L, Hou M, et al. Evaluation the combined diagnostic value of TAT, PIC, tPAIC, and sTM in disseminated intravascular coagulation: A multi-center prospective observational study. Thrombosis Research. janv 2019;173:20‑6.

10. Statz S, Sabal G, Walborn A, Williams M, Hoppensteadt D, Mosier M, et al. Angiopoietin 2 Levels in the Risk Stratification and Mortality Outcome Prediction of Sepsis-Associated Coagulopathy. Clin Appl Thromb Hemost. nov 2018;24(8):1223‑33.

11. Walborn A, Rondina M, Mosier M, Fareed J, Hoppensteadt D. Endothelial Dysfunction Is Associated with Mortality and Severity of Coagulopathy in Patients with Sepsis and Disseminated Intravascular Coagulation. Clin Appl Thromb Hemost. 1 janv 2019;25:107602961985216.

12. Higgins SJ, De Ceunynck K, Kellum JA, Chen X, Gu X, Chaudhry SA, et al. Tie2 protects the vasculature against thrombus formation in systemic inflammation. Journal of Clinical Investigation. 2 avr 2018;128(4):1471‑84.

13. Boscolo A, Campello E, Bertini D, Spiezia L, Lucchetta V, Piasentini E, et al. Levels of circulating microparticles in septic shock and sepsis-related complications: a case-control study. Minerva Anestesiol [Internet]. mai 2019 [cité 11 mars 2024];85(6). Disponible sur: https://www.minervamedica.it/index2.php?show=R02Y2019N06A0625

14. Zhang J, Xue M, Chen Y, Liu C, Kuang Z, Mu S, et al. Identification of soluble thrombomodulin and tissue plasminogen activator-inhibitor complex as biomarkers for prognosis and early evaluation of septic shock and sepsis-induced disseminated intravascular coagulation. Ann Palliat Med. oct 2021;10(10):10170‑84.

15. Wegrzyn G, Walborn A, Rondina M, Fareed J, Hoppensteadt D. Biomarkers of Platelet Activation and Their Prognostic Value in Patients With Sepsis-Associated Disseminated Intravascular Coagulopathy. Clin Appl Thromb Hemost. 14 févr 2021;27:1076029620943300.
