# Supplementary material for: Biomarkers of sepsis-induced coagulopathy: diagnostic insights and potential therapeutic implications
Source: Ann Intensive Care. 2025 Jan 17;15:12. doi: 10.1186/s13613-025-01434-2 (PMC11739444; doi:10.1186/s13613-025-01434-2)
Supplement: Supplementary file 5 — Supplementary Material 5 [file 13613_2025_1434_MOESM5_ESM.docx]

**Supplementary Table 5**. **Neutrophil extracellular traps in DIC**

| Reference | Study design | Population and number of patients | Markers | Results |
| --- | --- | --- | --- | --- |
| Delabranche et al. 2016 (1) | Prospective single center study | 20 patients with septic shock  10 with DIC  10 without DIC | Nucleosomes, NETs (DNA-MPO) | Circulating nucleosomes and DNA-MPO were increased in DIC patients (p<0.05) |
| Stiel et al. 2016 (2) | Prospective single center study | 100 patients with septic shock  35 DIC  65 without DIC | NEUT-SFL | A mean value of NEUT-SFL>57.3 AU had a sensitivity of 90.91% and a specificity of 80.60% for DIC diagnosis |
| Abrams et al. 2019 (3) | Prospective single center study | 341 adults in ICU  - 28 DIC* | NET | NET formation was significantly higher in patients with DIC (median, 50.0%; interquartile range, 25.0–88.0%) than in those without DIC (5.0%; 0.0–20.0%) (p< 0.0001) |
| Stiel et al. 2019 (4) | Prospective single center study | 9 patients  3 septic shock with DIC  3 septic shock without DIC  3 healthy donors | NEUT-SFL | NEUT-SFL was increased in DIC patients |
| Mao et al.  2021 (5) | Prospective single center study | 82 patients  34 patients with DIC | NET | NET formation was significantly higher in patients with DIC (p < 0.0001) |

AU: arbitrary units; DIC: disseminated intravascular coagulation; NA: not applicable; NS: non statistically significant; MPO: myeloperoxydase; NETs: neutrophil extracellular traps; NEUT-SFL: Neutrophil Side Fluorescence Light ; DIC: sepsis-induced coagulopathy; sTM: soluble thrombomodulin.

*DIC all causes: infection, leukemia, solid cancer, trauma, pregnancy, others.

1. Delabranche X, Stiel L, Severac F, Galoisy AC, Mauvieux L, Zobairi F, et al. Evidence of Netosis in Septic Shock-Induced Disseminated Intravascular Coagulation. Shock. mars 2017;47(3):313‑7.

2. Stiel L, Delabranche X, Galoisy AC, Severac F, Toti F, Mauvieux L, et al. Neutrophil Fluorescence: A New Indicator of Cell Activation During Septic Shock–Induced Disseminated Intravascular Coagulation. Critical Care Medicine. nov 2016;44(11):e1132‑6.

3. Abrams ST, Morton B, Alhamdi Y, Alsabani M, Lane S, Welters ID, et al. A Novel Assay for Neutrophil Extracellular Trap Formation Independently Predicts Disseminated Intravascular Coagulation and Mortality in Critically Ill Patients. Am J Respir Crit Care Med. 1 oct 2019;200(7):869‑80.

4. Stiel L, Mayeur-Rousse C, Helms J, Meziani F, Mauvieux L. First visualization of circulating neutrophil extracellular traps using cell fluorescence during human septic shock-induced disseminated intravascular coagulation. Thrombosis Research. nov 2019;183:153‑8.

5. Mao JY, Zhang JH, Cheng W, Chen JW, Cui N. Effects of Neutrophil Extracellular Traps in Patients With Septic Coagulopathy and Their Interaction With Autophagy. Front Immunol. 11 oct 2021;12:757041.
